# Response: Commentary: Pattern Recognition Proteins: First Line of Defense Against Coronaviruses

**DOI:** 10.3389/fimmu.2022.853015

**Published:** 2022-04-12

**Authors:** Carlos Alberto Labarrere, Ghassan S. Kassab

**Affiliations:** California Medical Innovations Institute, San Diego, CA, United States

**Keywords:** pattern recognition proteins, SP-D, glutathione, Nrf2, oxidative stress, innate immunity, SARS-CoV-2, COVID-19

## Introduction

De Pietro and Salzberg (1) emphasized the role of surfactant protein D (SP-D) in their commentary regarding the article “Pattern Recognition Proteins: First Line of Defense Against Coronaviruses A Commentary on Pattern Recognition Proteins: First Line of Defense Against Coronaviruses” by Labarrere and Ghassan (*Frontiers Immunology*, 2021, https://doi.org/10.3389/fimmu.2021.652252). We appreciate their comments about our article being informative and interesting and for making emphasis on the importance of innate immunity and particularly SP-D in the defense against multiple viral pathogens including SARS-CoV-2 (1). As noted, numerous studies highlighted the value of high serum SP-D levels upon development of severe COVID-19 disease (1, 2). As correctly pointed out, deficient pulmonary SP-D levels have been reported in severe COVID-19 and have been associated with increased risk of acute respiratory distress syndrome (1, 3, 4) and were also found in several chronic pulmonary diseases (5). These findings support the idea of using recombinant human pulmonary surfactant protein-D (rhSP-D) for the treatment of SARS-CoV-2 infection in order to reduce COVID-19 disease severity (4). The use of a shorter recombinant fragment of human SP-D (rfhSP-D) was able to bind to the S-protein of SARS-CoV-2 and reduce virus infection, replication, and cell entry (6, 7). Using rhSP-D dodecamers will add greater virus binding affinity, aggregation, and clearance capabilities against SARS-CoV-2 viruses. The use of SP-D molecules for the treatment of viral and other microbial diseases, as well as chronic diseases such as chronic obstructive pulmonary disease and asthma, and the SP-D deficiency in premature neonates requiring mechanical ventilation to reduce the risk for developing bronchopulmonary dysplasia is very promising (1).

## Discussion

Physicians and researchers, however, do not want to become overly simplistic regarding treatment of a complex, aggressive, and lethal disease such as COVID-19 caused by the SARS-CoV-2 virus. As recently noted, “In an ideal world, widespread access to and acceptance of vaccines to prevent SARS-CoV-2 infection could end the current pandemic; however, given imperfect vaccine uptake and ongoing emergence of variants, it is likely that SARS-CoV-2 will become endemic. Thus, there is a continued need for therapies that can be used early in the disease course to reduce the risk of disease progression, prevent transmission, and be widely distributed to meet global demand” (8). The rapid emergence of new SARS-CoV-2 variants (9, 10) with adaptive mutations in the spike protein can result in the persistence of the disease and certainly make necessary the use of a “multiple weaponry method” (2) to more effectively be able to reduce or perhaps eliminate the COVID-19 disease.

**Figure 1:**
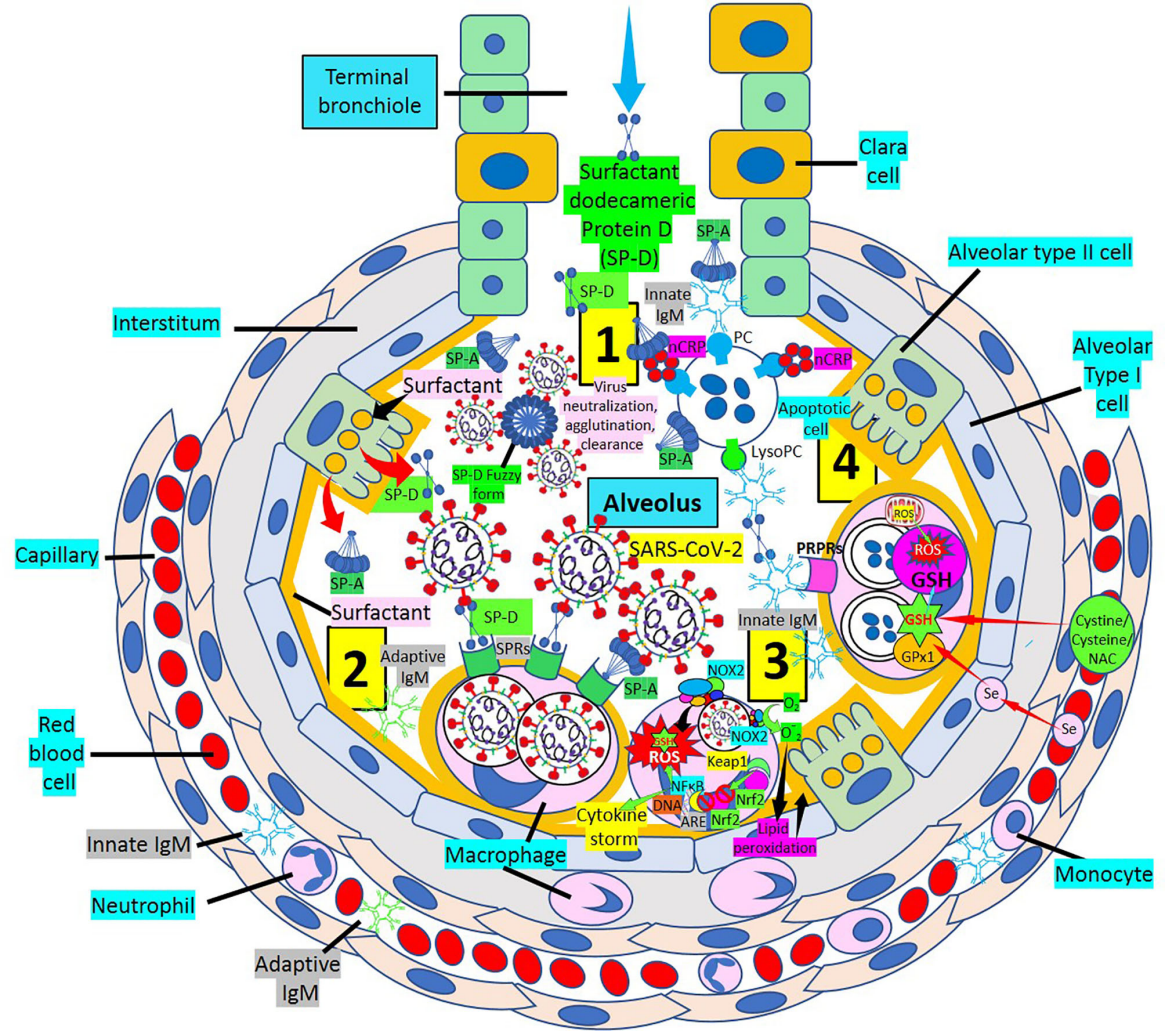
Multiweapon approach to treat SARS-CoV-2 infection and COVID-19 disease. [1] Inhaled dodecameric surfactant protein D (SP-D) treatment in association with endogenous SP-D (dodecamers, fuzzy form) and urfactant protein A (SP-A) facilitate neutralization of SARS-CoV-2 particles. [2] SARS-CoV-2 particles can be phagocytosed by alveolar macrophages *via* surfactant protein receptors (SPRs). [3] Macrophage nicotinamide adenine dinucleotide phosphate (NADPH) oxidase (NOX2) generates superoxide ( $O_{2}^{-}$ ) radicals increasing intracellular reactive oxygen species (ROS), causing lipid peroxidation and consuming GSH. Under oxidative stress and induced by ROS generation, nuclear factor erythroid 2 p45–related factor 2 (Nrf2) is released from repression mediated by Kelch-like ECH-associated protein 1 (Keap1) and translocated to the nucleus where it binds antioxidant response element (ARE) to activate antioxidant enzymes. Excessive and continuous oxidative stress inhibits Nrf2 translocation into the nucleus and enhances nuclear factor-kB (NF-κB) activation promoting inflammation, oxidative damage, and cytokine storm. Supplementing selenium and GSH precursors [cystine, cysteine, N-acetyl cysteine (NAC)] enhances GSH peroxidase-1 (GPx1) and GSH levels, respectively, increasing GSH activity and ROS neutralization. [4] Pattern recognition proteins (PRPs) such as native pentameric C-reactive protein (nCRP) bound to phosphorylcholine (PC), innate IgM/SP-A, CRP/SP-A, and SP-A participate in the removal of apoptotic cells, in association with SP-D and innate/adaptive IgM molecules *via* pattern recognition protein receptors (PRPRs). lysoPC, lysophosphatidylcholine.

Vaccines, neutralizing monoclonal antibodies, immune modulators, and antivirals are primary components of the treatment armamentarium (2, 8). Using dodecameric SP-D, SP-D inhibitory receptors (2), virus removal by other pattern recognition proteins [SP-A, pentameric C-reactive protein, innate immunoglobulin M (IgM) natural antibodies, Toll-like receptor inhibitors, and others] will facilitate reduction of lung disease severity in particular (**Figure 1**), and COVID-19 disease in general. By enhancing all lines of host defense that include soluble and cellular components of innate immunity, cellular components of both innate and adaptive immunity and soluble and cellular components of the adaptive immune system will certainly help humans defeat this pandemic killer (2). Considering the impact that cytokine storm and oxidative stress have during SARS-CoV-2 infection toward development of a severe disease, the concomitant administration of both nuclear factor erythroid 2 p45–related factor 2 (Nrf2) activators such as sulforaphane and glutathione (GSH) synthesis precursors such as N-acetyl cysteine, cysteine, and cystine can increase (a) nuclear Nrf2 translocation and antioxidant response element transcription and (b) synthesis of GSH, the most powerful antioxidant of the body, respectively (11–13), facilitating prevention against oxidative stress, inflammation, and cell and tissue damage in SARS-CoV-2 infection and COVID-19 disease. Replenishing the nutritional status of the host by (a) increasing vital amino acids such as cysteine and/or (b) providing GSH itself, through liposomal administration (14) to enhance GSH levels, and (c) supplementing selenium to improve selenium deficiency and facilitate selenoprotein (GSH peroxidases, thioredoxin reductases) expression (15) can inhibit oxidative stress, modulating inflammation and endothelial dysfunction. A multitreatment approach will likely have more successful outcomes against SARS-CoV-2 infection and COVID-19 disease than individual treatments.

## Author Contributions

All authors listed have made a substantial, direct, and intellectual contribution to the work and approved it for publication.

## Conflict of Interest

The authors declare that the research was conducted in the absence of any commercial or financial relationships that could be construed as a potential conflict of interest.

## Publisher’s Note

All claims expressed in this article are solely those of the authors and do not necessarily represent those of their affiliated organizations, or those of the publisher, the editors and the reviewers. Any product that may be evaluated in this article, or claim that may be made by its manufacturer, is not guaranteed or endorsed by the publisher.
